# Osthole Inhibits M1 Macrophage Polarization and Attenuates Osteolysis in a Mouse Skull Model

**DOI:** 10.1155/2023/2975193

**Published:** 2023-01-12

**Authors:** Feifei Wang, Peiming Yang, Tianhao Wan, Cong Liu, Yujuan Zhu, Xuzhuo Chen, Huanyan Liu

**Affiliations:** ^1^The Affiliated Tai'an City Central Hospital of Qingdao University, Tai'an, 271000 Shandong Province, China; ^2^Department of Stomatology, Xuzhou Central Hospital, Xuzhou, 221000 Jiangsu Province, China; ^3^Shanghai Jiao Tong University School of Medicine, Shanghai 200011, China; ^4^Department of Stomatology, Tai'an Second Hospital of Traditional Chinese Medicine, Tai'an, 271000 Shandong Province, China; ^5^Shanghai Key Laboratory of Stomatology, Department of Oral Surgery, Ninth People's Hospital, College of Stomatology, Shanghai Jiao Tong University School of Medicine, No. 639, Zhi Zao Ju Rd, Shanghai 200011, China

## Abstract

Excessive bone resorption due to increased inflammatory factors is a common feature of inflammatory lytic bone diseases. This group of diseases is effectively treated with drugs. In recent years, many studies have reported that traditional Chinese medicine herbs have substantial effects on inflammation, osteoclast differentiation and maturation, and bone destruction. Herein, we investigated the effects of osthole (OST) on lipopolysaccharide- (LPS-) induced macrophage polarization, inflammatory responses, and osteolysis. In vitro, we used immunofluorescence and quantitative real-time polymerase chain reaction assays to confirm whether bone marrow-derived macrophages showed an increased expression of inflammatory factors, such as interleukin-6, iNOS, CCR7, and CD86, in the presence of LPS. However, we found that such expression was suppressed and that the M2 macrophage expression increased in the presence of OST. OST reduced LPS- and RANKL-induced intracellular reactive oxygen species production in the bone marrow-derived macrophages. Further, it potently suppressed osteoclast differentiation and osteoclast-specific gene expression by suppressing the P38/MAPK and NF-*κ*B pathways. Consistent with the in vitro observations, OST greatly ameliorated LPS-induced bone resorption and modulated the ratio of macrophages at the site of osteolysis. Taken together, OST has great potential for use in the management of osteolytic diseases.

## 1. Introduction

“Bone remodeling” occurs throughout a person's life [1], in which osteoblasts (OBs) and osteoclasts (OCs) play an essential role. OBs play a central role in bone formation by forming new bone matrix proteins and differentiating into osteocytes, whereas OCs are the only cells with bone resorption function in the body. However, inflammatory OCs are often abnormally active and cause osteolysis due to inflammation and hormonal changes, including rheumatoid arthritis (RA) [2], chronic periodontal disease [3], osteomyelitis [4], and peri-implantitis [5]. The search for drugs with fewer toxic side effects is an effective means to improve the treatment outcomes of these diseases.

Bacteria generated at the interface of implants and the bones can interfere with the aggregation of bone marrow-derived macrophages (BMMs). Thus, they further promote the differentiation of BMMs into OCs and release large amounts of proinflammatory factors, such as interleukin- (IL-) 1, tumor necrosis factor-*α*, and IL-6 [6, 7]. Macrophages exhibit different phenotypes, mainly the proinflammatory type and anti-inflammatory M2 type, depending on the changes in the bone immune microenvironment. An increase in M1 macrophage production is observed in many inflammatory bone diseases, for example, in periodontitis, there is an M1 polarization of the macrophage phenotype, a phenotype that leads to the loss of periodontal attachment and resorption of alveolar bone [8]. In RA, proinflammatory factors secreted by M1 macrophages exacerbate the symptoms, whereas anti-inflammatory factors secreted by M2 macrophages alleviate such. In addition, the anti-inflammatory cytokines IL-10 and transforming growth factor-*β*, which are secreted by M2 macrophages, can be used to treat RA [9, 10]. When M1 macrophages dominate implants, there are usually inflammation around the implants and imbalance in bone metabolism, which eventually lead to implant failure [11]. These findings demonstrate the detrimental effects of the proinflammatory M1 phenotype in inflammatory bone diseases; therefore, investigators have focused on macrophage polarization in the hope of finding effective targets for the treatment of inflammatory bone diseases.

BMMs differentiate into OCs without the induction of M-CSF and RANKL ([12, 13]. M-CSF mainly maintains the survival, proliferation, and maturation of monocyte-macrophages, induces BMMs to express RANK receptors, and becomes OC precursor cells. Body cells are released by OBs and fibroblasts, and during inflammatory responses, RANKL from activated T and B lymphocytes binds to RANK on OC precursor cells, activating Toll-like receptors, mainly tumor necrosis factor receptor-associated fraction 6, further activating the AKT, NF-*κ*B, and MAPK pathways and promoting OC formation.

According to the literature ([14–16], osthole (OST) has anti-inflammatory, thrombotic, antitumor, and antiallergic effects. OST ameliorated kidney damage by inhibiting KHK activity, preventing oxidative stress via nuclear factor erythroid 2-related factor (Nrf2) activation, and reducing renal lipotoxicity [17]. OST increased the protein expression of p38 mitogen-activated protein kinase (MAPK) and type I collagen and enhances the differentiation and maturation of osteoblasts in vitro [18]. However, the effect of OST on macrophage polarization and osteoclast differentiation has not been extensively studied. Therefore, we investigated the effects of OST on lipopolysaccharide- (LPS-) induced M1 macrophage polarization, as well as the resulting osteoprotective effects.

## 2. Materials and Methods

### 2.1. Reagents

OST (S233701) was purchased from Selleck (Houston, TX) and dissolved in dimethyl sulfoxide at a concentration of 50 mM as a stock solution; *α* minimal essential medium (*α*-MEM) from Basalmedia (Shanghai, China); fetal bovine serum from Gibco BRL (Sydney, NSW, Australia); penicillin from Gibco BRL (Gaithersburg, MD); cell counting kit-8 (CCK-8) from Dojindo Molecular Technology (Kumamoto, Japan); tartrate-resistant acid phosphatase (TRAP) staining solution from Sigma-Aldrich (St. Louis, MO); recombinant RANKL and M-CSF from R&D Systems (Minneapolis); primary antibodies against P38, JNK, ERK1/2, P65, CD206, iNOS, and GAPDH and secondary antibodies conjugated with fluorescent dye from CST (Danvers); Osteo Assay Stripwell 96 Plates from Corning; and all other reagents from Sigma-Aldrich.

### 2.2. Cell Culture

The BMMs were prepared as described previously [19]. Briefly, the BMMs were extracted from the femurs and tibias of 6-week-old C57/BL6 male mice. Isolated cells were cultured in complete *α*-MEM (*α*-MEM supplemented with 10% fetal bovine serum, 100 U/mL penicillin/streptomycin, and 30 ng/mL M-CSF). The BMMs were obtained by culturing the cells in a humid environment containing 5% CO_2_ at 37°C for 5 days.

### 2.3. Cell Viability Assay

To assess whether OST was toxic to the BMMs, the BMMs were seeded at a density of 1 × 10^4^ cells/well and supplied with complete *α*-MEM, M-CSF (30 ng/mL) and increasing concentrations of OST (0, 1.5, 3, 6.25, 12.5, 25, 50, 100, 200, and 400 *μ*M). Following treatment with OST, the cells were incubated for 24, 48, and 72 h in a 96-well plate. After treatment, 10 *μ*L of CCK-8 solution was added to each well and incubated for 2 h, and the absorbance at 450 nm was measured using an enzyme marker. The effect of OST on cell viability was expressed as percentage cell viability, with the viability of the control cells set to 100%, and the half-inhibitory concentration was calculated for 48 and 72 h.

### 2.4. Immunofluorescence Staining

The BMMs (2 × 10^5^ cells/well) were plated in confocal culture plates (Seahorse Bioscience, North Billerica, USA) and allowed to attach overnight. The cells were treated with 100 ng/mL LPS to induce M1 polarization. During polarization induction, different concentrations of OST (6.25, 12.5, and 25 *μ*M) were added for 24 h. The BMMs were fixed in paraformaldehyde with 0.1% Triton X-100 (Sigma, USA). Goat serum (4%) was used to block nonspecific binding. The cells were then incubated overnight with iNOS and CD206 primary antibodies (CST, USA; dilution 1 : 200). The fluorescent secondary antibodies were incubated at room temperature for 1 h. The cells were stained with 4,6-diamidino-2-phenylindole for 5 min and observed under a fluorescence microscope.

### 2.5. Reactive Oxygen Species (ROS) Assay

Intracellular ROS formation was detected using a DCFH-DA probe. The BMMs were inoculated in 96-well plates (black wall/clear bottom) at a density of 2 × 10^5^ cells/well and then stimulated with M-CSF treatment alone or M-CSF, LPS, and different concentrations of OST (6.25, 12.5, and 25 *μ*M) for 24 h. DCFH-DA and Hoechst (dilution, 1 : 1000) were loaded in serum-free *α*-MEM for 20 min at 37°C. Fluorescence intensity was measured using a fluorescence microscope (DMI8; Leica) and a multifunctional microplate reader (Infinite F500; Monidorf Dicken, Switzerland).

### 2.6. Quantitative Real-Time Polymerase Chain Reaction (qRT-PCR)

The BMMs were inoculated in 6-well plates at a density of 4 × 10^5^ cells/well, and after the cells were plastered, the experimental and control groups were treated with the same immunofluorescence, with 3 subwells for each group. Total RNA was extracted from the macrophages using the TRIzol method, and the purity and concentration of the extracted RNA were tested using a NanoDrop 2000/2000c spectrophotometer. The extracted RNA was reverse-transcribed into cDNA using a PrimeScript Reverse Transcription Kit. The PCR reaction parameters were as follows: 95°C for 5 s, 60°C for 30 s, 40 cycles, using GAPDH as the internal reference. The 2 − ΔΔCT method was used to calculate the relative gene expression. The primer sequences used are listed in Table 1.

### 2.7. In Vitro Osteoclastogenesis Assay

To study the effect of OST on OB differentiation, we stimulated the M-CSF-dependent BMMs (1 × 10^4^ cells/96 − well plate well) with 50 ng/mL RANKL for 5 days, with or without different concentrations of OST (0, 6.25, 12.5, and 25 *μ*M). The medium containing M-CSF, RANKL, and OST was replenished every other day until OBs formed in the control wells with RANKL only (day 5). The cells were then fixed in 4% paraformaldehyde for 20 min and stained with the TRAP staining solution for 1 h at 37°C. Digital images were acquired using a light microscope (Olympus). The number of TRAP-positive OCs containing three or more nuclei and the area of cell spread was analyzed using the ImageJ software (NIH, Bethesda, MD).

### 2.8. Intracellular ROS Measurement

The BMMs were inoculated at a density of 1 × 10^4^ in 96-well plates (black wall/clear bottom) and then subjected to M-CSF treatment alone or M-CSF, RANKL, or OST (25 *μ*M) stimulation for 48 h to maintain the macrophages or generate pre-OCs, respectively. The detection method was the same as that described in section 2.5.

### 2.9. qRT-PCR Analysis of OC-Related Gene Expression

After the BMMs were counted, they were inoculated in 6-well plates at a ratio of approximately 4 × 10^5^ cells/well. M-CSF (30 ng/mL) was routinely added to the medium, and the medium containing 50 ng/mL RANKL and different concentrations of OST (6.25, 12.5, and 25 *μ*M) was replaced after 24 h. The medium was changed every other day, and the cells were treated with drugs for 5 days, followed by RNA extraction, reverse transcription, and PCR amplification, as described in Section 2.6. The mouse primer sequences used are listed in Table 2.

### 2.10. Western Blot Analysis

To determine the effect of OST on RANKL-induced signaling, we measured the total cellular proteins at different time points. The proteins were extracted from the BMMs treated with 25 *μ*M OST for 8 h, followed by stimulation with RANKL (50 ng/mL) for 10, 20, 30, and 60 min (short time course). The untreated cells were considered as mock controls (0 time point). The cultured cells were lysed with RIPA lysis buffer containing phosphatase and protease inhibitors for 10 min to obtain the total value. The protein concentration was determined using bicinchoninic acid assay. Thirty micrograms of the total cellular proteins was separated by 10% sodium dodecyl sulfate-polyacrylamide gel electrophoresis and then transferred to polyvinylidene difluoride membranes. After blocking in 5% (*w*/*v*) skim milk for 1 h, the primary antibodies (GAPDH, 1 : 1000; P-P65, 1 : 1000; P65, 1 : 1000; P-ERK, 1 : 1000; ERK, 1 : 1000; P-P38, 1 : 1000; P38, 1 : 1000; P-JNK, 1 : 1000; and JNK, 1 : 1000) were incubated at 4°C overnight. Thereafter, the secondary antibodies were incubated for 1 h at room temperature, and the fluorescence signals were determined using an Odyssey imaging system (Li-Cor, Lincoln, NE).

### 2.11. LPS-Induced Calvarial Osteolysis Mice Model

All animal experiments were approved by the Ethics Committee of Shanghai Jiao Tong University School of Medicine. Twenty C57BL/6 female mice (age: 6 weeks, weight: 20 ± 0.10 g) were randomly divided into four groups (five mice per group): the sham-operated (injected with PBS), the LPS group (injected with 10 mg/kg LPS and PBS), the LPS+low-dose OST group (injected with LPS and 1.5 mg/kg OST), and the LPS+high-dose OST group (injected with LPS and 7.5 mg/kg OST). After anesthetizing the mice, we implanted a gelatin sponge (4 × 4 × 2 mm) soaked with PBS or LPS (200 *μ*g) in the calvaria. In the groups, PBS and OST were intraperitoneally injected every other day over a 14-day period. No adverse effects or deaths occurred in any mouse group during the experimental period. At the end of the experiment, the mice were sacrificed, and the dissected cranial bones were fixed in 4% paraformaldehyde for 48 h for subsequent analysis.

### 2.12. Micro-CT Scanning

A micro-CT scanner (Skyscan 1076; Bruker, Billerica, MA; resolution: 9 *μ*M; X-ray source: 29 kv/175 uA; exposure time: 300 ms) was used for calvarial scanning. A region of interest was defined as a square around the resorption area for bone volume analysis (Wedemeyer et al., 2007).

### 2.13. Histological Staining and Histomorphometric Analysis

The calvarial samples were decalcified in 10% EDTA (pH: 7.4) for 2 weeks, and 4 *μ*M tissue sections were cut into sections. The sections were stained with hematoxylin and eosin and TRAP. Digital images were acquired using an Axio Scope A1 light microscope (ZEISS, Germany). The number of OCs was quantified using the ImageJ software.

### 2.14. Tissue Immunofluorescence Staining

Antigen repair was first performed by placing the sections in 0.1 mol/L sodium citrate repair solution, heating them in a microwave oven for antigen repair, and cooling them for 30 min. The sections were soaked in 10% goat serum for 1 h to remove nonspecific staining; the goat serum was discarded; and the sections were incubated with rabbit anti-mouse iNOS and CD206 at a 1 : 500 dilution and incubated overnight at 4°C. After incubation with the primary antibody, the cells were incubated with the fluorescent secondary antibody (1 : 1000) for 1 h, protected from light, sealed, and dried at room temperature, and images were collected under a fluorescence microscope.

### 2.15. Statistical Analysis

GraphPad Prism 8.0 was used to process and analyze the data obtained from the experiments. The *t*-test was used between two samples, and one-way ANOVA was used between multiple samples, with *p* < 0.05, *p* < 0.01, and *p* < 0.001 indicating significant differences.

## 3. Results

### 3.1. OST Exerted No Cytotoxic Effects at Low Concentrations

**Figure 1:**
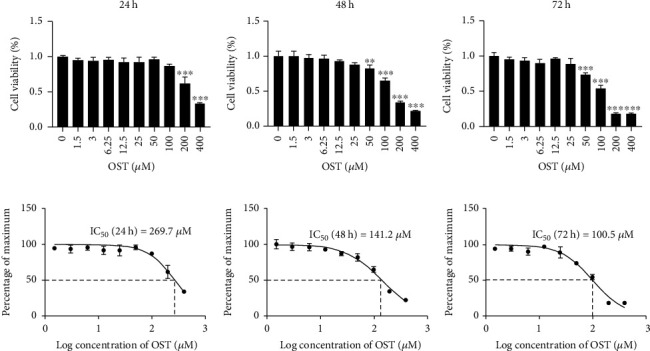
Effect of OST on the cell viability of the BMMs in the CCK-8 assay. (a) The proliferation and viability of the BMMs were assessed using the CCK-8 assay following incubation with indicated concentrations of OST for 24, 48, and 72 h. (^∗^*p* < 0.05, ^∗∗^*p* < 0.01, ^∗∗∗^*p* < 0.001). (b) The half-inhibitory concentration of OST in the BMMs was 269.7 *μ*M at 24 h, 141.2 *μ*M at 48 h, and 100.5 *μ*M at 72 h.

The CCK-8 analysis showed that low concentrations of OST were not cytotoxic to the BMMs; 25 *μ*M and lower concentrations did not exert cytotoxic effects at 24, 48, and 72 h. The half-inhibitory concentration of OST was 269.7 *μ*M at 24 h, 141.2 *μ*M at 48 h, and 100.5 *μ*M at 72 h (Figure 1(b)). We selected three groups that were not toxic to the cells (6.25, 12.5, and 25 *μ*M) for the subsequent experiments.

### 3.2. OST Inhibited ROS Production in the LPS- and RANKL-Induced BMMs

**Figure 2:**
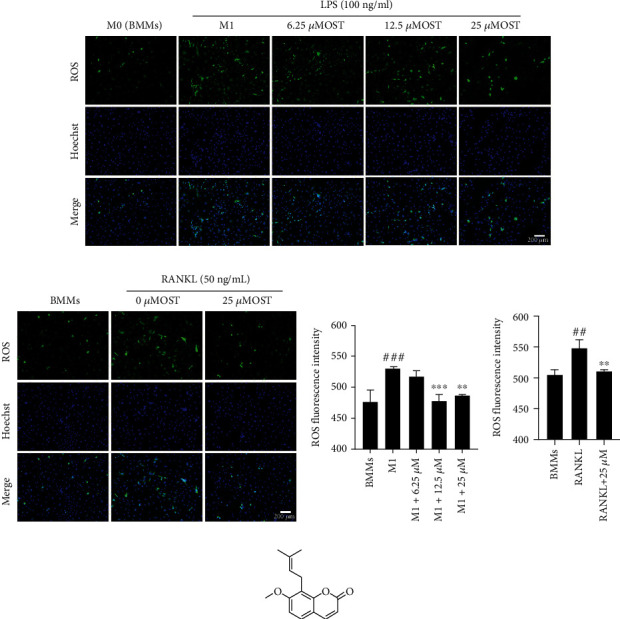
Effect of OST on ROS generation. (a) Production of intracellular ROS in each experimental group after 24 h of LPS and OST treatments. (b) Production of intracellular ROS in each experimental group after 48 h of RANKL and 25 *μ*M OST treatments. (c, d) A multifunctional microplate reader was used to measure and calculate the fluorescence intensity of ROS in each experimental group. (e) Chemical structure of OST. (^#^compared with the BMM group, ^∗^compared with the M1 or RANKL group, ^###^*p* < 0.001, ^∗^*p* < 0.05, ^∗∗^*p* < 0.01, ^∗∗∗^*p* < 0.001). Data are expressed as median and interquartile range, *n* = 3.

**Figure 3:**
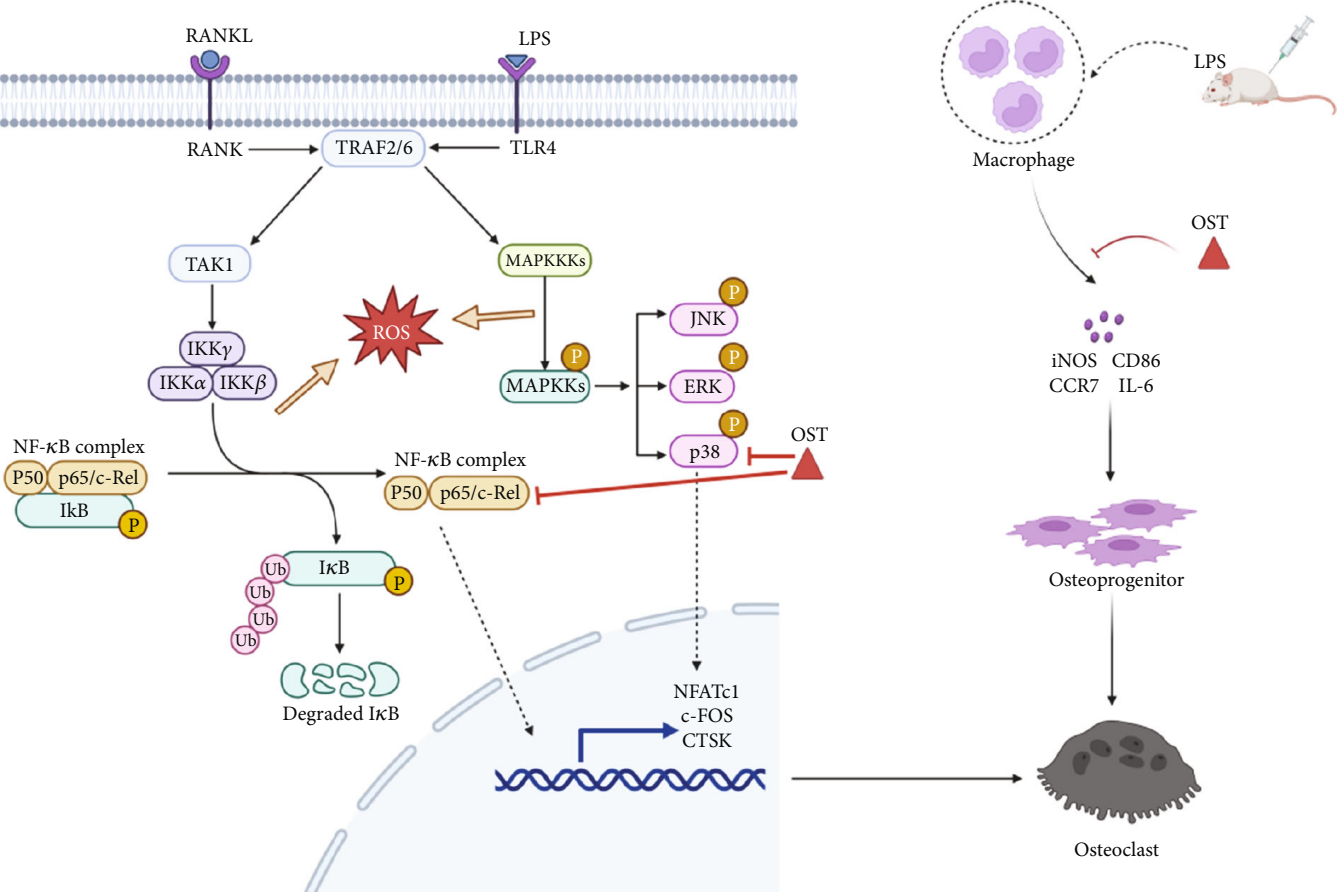
Schematic representation of the molecular mechanism of the article (created with http://BioRender.com).

After induction with 100 ng/mL LPS and 50 ng/mL RANKL, the ROS expression in the BMMs increased (Figures 2(a) and 2(b)). OST inhibited ROS production in the BMMs at different degrees, and the higher the OST concentration, the weaker the DCFH-DA fluorescence intensity (Figures 2(c) and 2(d)). The DCF fluorescence intensity in each well was evaluated and quantified. OST treatment markedly reduced intracellular ROS production compared with LPS or RANKL treatment alone. The fluorescence intensity was also significantly lower in the presence of OST than in 520(*p* < 0.05). In summary, our findings demonstrate that OST acts as an inhibitor to reduce ROS signaling pathways during ROS production in the LPS- and RANKL-induced BMMs (Figure 3).

### 3.3. OST Inhibited LPS-Induced Polarization of the M1 Macrophages

**Figure 4:**
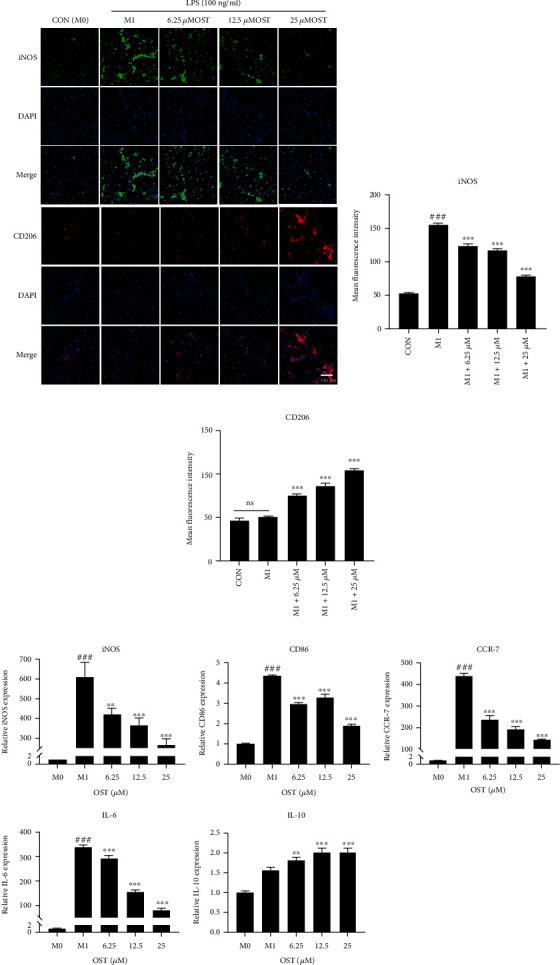
Effect of the different concentrations of OST (6.25, 12.5, and 25 *μ*M) on the LPS-induced M1-type polarization in the BMMs. (a) Immunofluorescence detection of the effect of the different concentrations of OST on iNOS and CD206 expression. (b, c) The mean fluorescence intensities of iNOS and CD206 were statistically calculated for each group. (d) Effect of the different concentrations of OST on the inflammation-related genes. Treated with different concentrations of OST for 24 h. Gene expression was analyzed by real-time PCR. mRNA expression levels were normalized relative to the expression of GAPDH (^#^compared with the control (M0) group, ^∗^compared with the M1 group, ^###^*p* < 0.001, ^∗^*p* < 0.05, ^∗∗^*p* < 0.01,  ^∗∗∗^*p* < 0.01). Data are expressed as median and interquartile range, *n* = 3.

In this experiment, the effect of different concentrations of OST on the expression of the LPS-induced M1 macrophage surface marker iNOS and M2 macrophage surface marker CD206 was examined using immunofluorescence. As shown in Figure 4(a), the expression of iNOS was elevated after LPS treatment in the addition group compared with that in the control group; the degree of decrease differed between the addition and LPS only groups (M1 group), and the most obvious decrease was observed in the M1+25 *μ*mol/L OST group (Figure 4(b)). In contrast, CD206 showed an opposite trend, with no significant change in its expression in the M1 group compared with that in the control group; however, the degree of elevation differed between the dosing and M1 groups, and the M1+25 *μ*mol/L OST group showed the strongest CD206 fluorescence intensity (Figure 4(c)).

We then employed qRT-PCR to evaluate the effect of OST on the LPS-induced inflammation-related genes in the BMMs. LPS induced the polarization of the BMMs to the M1 type, and the expression of the proinflammatory genes (iNOS, IL-6, CCR7, and CD86) significantly increased (*p* < 0.001). Meanwhile, the expression of iNOS, CD86, IL-6, and CCR7 significantly decreased after the addition of the different concentrations of OST compared with those after LPS treatment (*p* < 0.01). The iNOS, IL-6, and CCR7 levels decreased in a concentration-dependent manner with OST. In contrast, the transcript levels of IL-10, an inflammatory factor, increased at different degrees in the dosing group, and the most significant increase was observed in the 25 *μ*M OST group. These results showed that OST inhibited the expression of the M1 proinflammatory factors, and the inhibitory effect was positively correlated with the concentration and increased the expression of the M2 proinflammatory factors to some extent.

### 3.4. OST Reduced RANKL-Induced OC Differentiation

**Figure 5:**
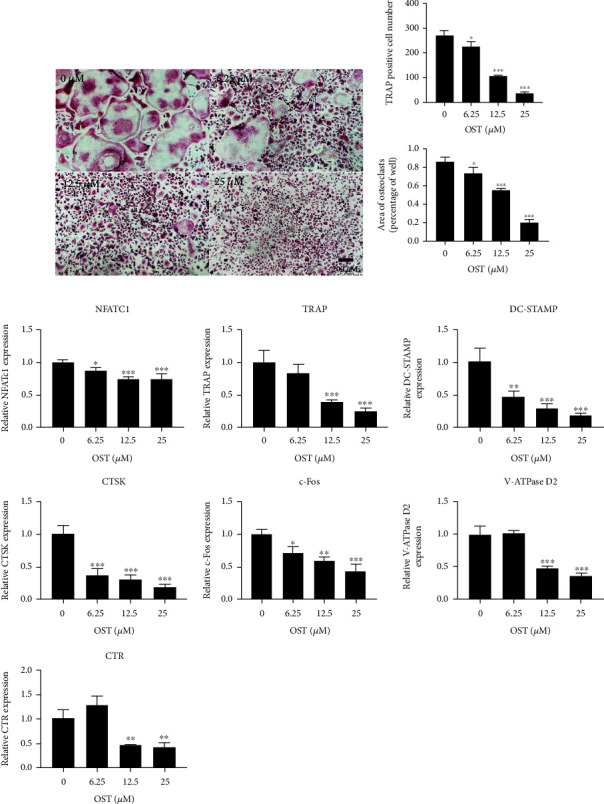
OST inhibited RANKL-induced osteoclast formation in vitro. (a) Representative images of TRAP-stained multinucleated osteoclasts treated with OST. (b, c) The number and size (in terms of area) of TRAP-positive multinucleated osteoclasts with three or more nuclei were quantified. (d) OST suppressed RANKL-induced expression of the osteoclast marker genes. Real-time quantitative PCR was conducted using RNA extracted from the BMM-derived osteoclasts treated with the indicated concentrations of OST for 5 days. The expression of NFATc1, c-Fos, DC-STAMP, V-ATPase d2, TRAP, CTR, and CTSK was normalized to the expression of the housekeeping gene GAPDH. (^∗^*p* < 0.05, ^∗∗^*p* < 0.01, ^∗∗∗^*p* < 0.01). Data are expressed as median and interquartile range, *n* = 3.

To further investigate whether OST could inhibit OC differentiation, we used 50 ng/mL RANKL to induce OC precursor cells to differentiate into OCs. The TRAP staining kit could stain OC-associated enzymes purple red under acidic conditions, and under TRAP staining, the cells larger than three nuclei and stained purple red were OCs. As shown in Figure 5(a), the precursor cells fused into one large vacuole-like cell with purplish red coloration upon stimulation with RANKL, and the number and area of OC formation increased. Meanwhile, the same RANKL stimulation with the addition of the different concentrations of OST significantly decreased the number of colored multinucleated cells, and the number and area of TRAP-positive cells showed a concentration-dependent decrease (Figures 5(b) and 5(c)); 25 *μ*M OST inhibited the differentiation of OBs most significantly.

The expression of the OC-specific genes was analyzed using qRT-PCR. We investigated the results of the RANKL and OST interventions for 5 days. The expression of the OC-specific genes, including NFATc1, CTR, CTSK, V-ATPase d2, TRAP, c-Fos, and DC-STAMP, increased in the control group after RANKL stimulation; conversely, the expression was significantly suppressed partly in a dose-dependent manner after OST treatment at the three concentrations of 6.25, 12.5, and 25 *μ*M (Figure 5(d)). NFATc1 played an important role in OC differentiation; c-Fos assisted in NFATc1 activation; and CTSK, c-Fos, TRAP, and DC-STAMP decreased the transcript levels in a dose-dependent manner. These results collectively showed that OST inhibited OC differentiation by downregulating the OC marker genes during OC differentiation, with 25 *μ*M OST yielding the most effective inhibition.

### 3.5. OST Inhibited the Phosphorylation of the NF-*κ*B and MAPK Pathway-Related Proteins

**Figure 6:**
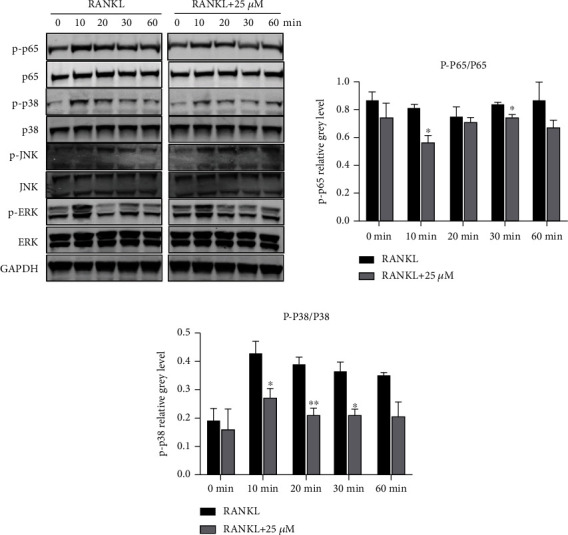
OST inhibited the RANKL-induced activation phosphorylation of NF-*κ*B and P38/MAPK. (a) OST impaired the RANKL-induced phosphorylation of P65 and P38 but not of P-JNK and P-ERK. (b, c) Quantitative densitometric analysis of P-P65 and P-P38 phosphorylation normalized to the total P65 and P38 expression. (^∗^*p* < 0.05, ^∗∗^*p* < 0.01, ^∗∗∗^*p* < 0.001). Data are expressed as median and interquartile range, *n* = 3.

To further investigate the mechanism related to OST inhibition of osteolysis, we detected the protein expression in the NF-*κ*B and MAPK pathways using western blotting and analyzed the protein expression in the RANKL and RANKL+25 *μ*M OST groups via short-term stimulation with RANKL. As shown in Figure 6(a), the expression of P-P38, P-P65, and P-ERK increased and gradually decreased over time upon stimulation with RANKL for 10 min, indicating that the addition of RANKL stimulated the activation of the NF-*κ*B and MAPK pathways in a short period. The RANKL+25 *μ*M OST group showed a different protein expression at 10 min. The P-P38 and P-P65 expression decreased in the RANKL+25 *μ*M OST group compared with that in the unspiked group, whereas the P-ERK and P-JNK expression did not differ between them. The expression of the related proteins in the two groups was analyzed and counted, as shown in Figures 6(b) and 6(c). The expression of P-P38 in the RANKL+25 *μ*M OST group decreased at 10, 20, and 30 min compared with that in the RANKL-plus alone group (*p* < 0.05); the expression of P-P65 also decreased at 10 and 30 min in the RANKL+25 *μ*M OST group compared with that in the RANKL-plus alone group (*p* < 0.05). Taken together, OST exerted its antibreakage effect by inhibiting the NF-*κ*B and P38/MAPK pathways activated by RANKL (Figure 3).

### 3.6. OST Suppressed LPS-Induced Calvarial Osteolysis In Vivo

**Figure 7:**
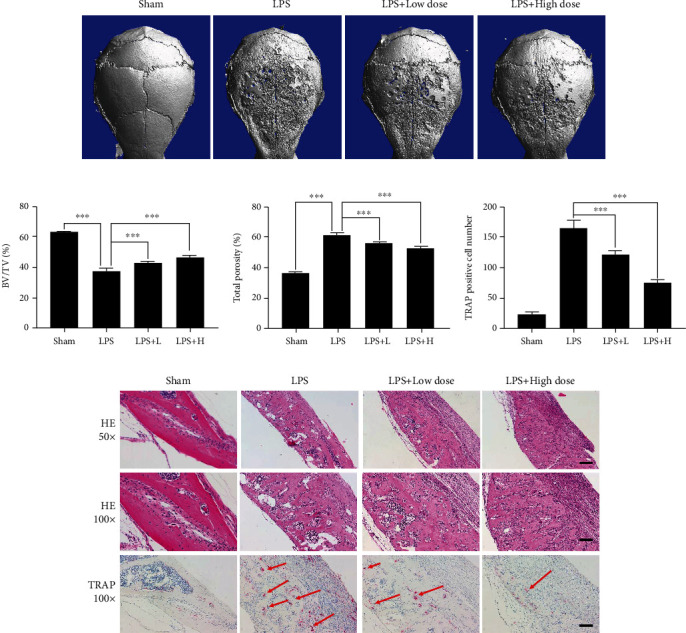
OST protected mice against LPS-induced inflammatory osteolysis in vivo. (a) Representative three-dimensional micro-CT reconstructions of murine calvaria from each treatment group. Collagen sponges soaked with PBS (sham) or LPS (10 mg/kg body weight) were implanted over the mouse calvarial bones, and PBS or OST (1.5 or 7.5 mg/kg) was injected subcutaneously over the sagittal midline suture of the calvaria over a 14-day period. Scale bar = 1 mM. (b, c) Morphometric analysis of the percentage of bone volume to tissue volume (BV/TV, %) and percentage of total porosity was conducted. (d) Histomorphometric analysis of the number of TRAP-positive osteoclasts in the calvarial bone sections. (^∗^*p* < 0.05, ^∗∗^*p* < 0.01, ^∗∗∗^*p* < 0.001). (e) Representative hematoxylin and eosin- (×50 or 100) and TRAP- (×100) stained sections from each treatment group. Scale bar = 100 *μ*M(×50), 200 *μ*M(×100). Data are expressed as median and interquartile range, *n* = 3–6.

We further investigated the potential application of OST in the treatment of inflammatory bone loss. We used LPS-induced inflammatory osteolysis in a mouse model of cranial bone injury. Gelatin sponge blocks impregnated with PBS or LPS (10 mg/kg) were implanted into the skulls of the mice to induce bone loss. The mice in each treatment group were injected subcutaneously with PBS or OST (low dose of 1.5 mg/kg or high dose of 7.5 mg/kg) into the sagittal suture of the skullcap on alternate days for 14 days. The 3D-CT reconstruction is shown in Figure 7(a). The skull surface of the LPS group showed extensive bone erosion, with deep and large resorption pits. In contrast, bone loss was significantly reduced in the mice treated with OST. Morphometric analysis showed a significant improvement in the bone volume compared with that after LPS treatment. There was a modest increase in the BV/TV (Figure 7(b)) compared with that in the sham-operated group. Similarly, bone porosity was reduced in the OST-treated animals compared with that in the LPS-treated animals (Figure 7(c)).

Histological and histomorphometric analyses of the cranial sections were performed to further confirm the osteoprotective effects of OST. The hematoxylin and eosin- and TRAP-stained sections showed extensive osteolytic destruction of cranial bone tissue in the LPS group, which contained a large number of TRAP-positive OCs on the surface of the osteolytically destroyed cranial bone (Figure 7(d)). Osteolytic bone loss and the number of OCs significantly decreased by approximately 26% and 55% in the low- and high-dose OST treatment groups, respectively (Figure 7(e)).

### 3.7. OST Decreased the Expression of iNOS and Increased the Expression of CD206 at the Site of LPS-Induced Skull Lysis

**Figure 8:**
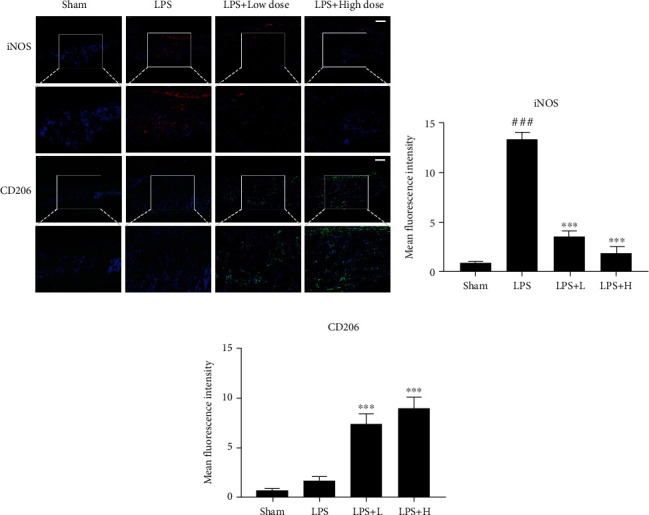
(a) Effect of OST on macrophage polarization at the site of LPS-induced cranial osteolysis in the mice. Scale bar = 100 *μ*M. (b,c) Mean fluorescence intensity of iNOS and CD206. (^###^compared with sham, ^∗^compared with LPS, ^∗^*p* < 0.05, ^∗∗^*p* < 0.01, ^∗∗∗^*p* < 0.001). Data are expressed as median and interquartile range, *n* = 3.

To further investigate whether OST treatment also has a role in regulating macrophages in vivo, we investigated the expression of the related proteins in the tissues using immunofluorescence and observed the expression of M1 macrophage marker protein iNOS and M2 macrophage marker protein CD206 in the tissues surrounding the skull lysis of the mice. As shown in Figure 8(a), a large amount of red fluorescence was visible at the edge of the tissue and inside the tissue, and the expression of iNOS increased (*p* < 0.05) in the LPS group. The expression of iNOS decreased in both the high- and low-dose treatment groups and the red fluorescence was almost invisible in the high-dose group (*p* < 0.05). The green fluorescence indicated the expression of CD206 and compared with the LPS group, the high- and low-dose treatment groups showed an increased area of green staining and increased CD206 expression (*p* < 0.05). Therefore, OST decreased the expression of iNOS and increased the expression of CD206 at the site of LPS-induced cranial lysis in the mice.

## 4. Discussion

Chronic inflammatory bone diseases, such as chronic periodontitis and RA, are autoimmune diseases that seriously affect human health. Currently, the main method of treating inflammatory bone diseases is pharmacological treatment, which includes NSAIDs, bisphosphonates, denosumab, tissue proteinase K inhibitors, and anabolic agents [20, 21]). However, the side effects and high price of these drugs limit their clinical application. Therefore, the development of new drugs for inflammatory bone diseases is imperative [22]. Currently, traditional Chinese medicine is gradually gaining attention for regulating the balance of bone metabolism because of its advantages of multiple pathways, multiple targets, and few toxic side effects.

Macrophage polarization during osteolysis is common. One study reported that in synovial joints under physiological conditions, macrophages remain quiescent along with fibroblasts, and when arthritis occurs, macrophages polarize to the proinflammatory M1 type according to changes in the microenvironment [23]. During the progressive phase of RA, most infiltrating macrophages are under the M1 type; the production of inflammatory cytokines, such as IL-6 and IL-23, can polarize primitive T cells into pathogenic Th17 cells, which promotes the production of OCs and thus initiates the process of osteolysis [24]. When macrophages polarize to the anti-inflammatory M2 type, they secrete IL-10 and Arg-1 anti-inflammatory factors, and IL-10, an important immunomodulatory factor, can inhibit osteoclastogenesis and play an osteoprotective role in periodontal disease [24, 25]. Natural compounds, such as kinsenoside, quercetin, and fargesin, have been found to modulate macrophage polarization and suppress OC activity, highlighting their potential as alternative therapeutics for disorders of bone metabolism [26–28].

LPS activates macrophages and polarizes them toward the M1 type, which is the classical induction method for M1 macrophages [29]. OST has an inhibitory effect on inflammation; however, it is not clear whether it affects the polarization of macrophages. In this experiment, the effect of OST on macrophage behavior was investigated by inducing BMMs toward M1 using 100 ng/mL of LPS. Using immunofluorescence and qRT-PCR, we found that LPS induced polarization of the BMMs toward the M1 type and increased the production of the proinflammatory factors. The decrease in the production of the inflammatory factors was most dominant after the addition of 25 *μ*M OST, indicating that OST could reduce the proportion of M1 macrophages and increase the proportion of M2 macrophages to some extent. Moreover, qRT-PCR showed that the expression of the proinflammatory factor IL-10 also increased to some extent after the addition of OST; however, it has also been reported in the literature that OST can attenuate the infiltration of M2 macrophages around pancreatic cancer tumors to inhibit the progression [30], which may be attributed to the fact that the inflammatory microenvironment is fundamentally different from the tumor microenvironment, and a different microenvironment leads to very different changes in macrophages.

There is a strong link between inflammation and osteolysis, with most inflammatory factors primarily activating the RANK/RANKL/OPG signaling pathway, which consequently triggers the promotion of OC differentiation, leading to an imbalance in bone remodeling and bone loss.

In our study, OST was found to have an inhibitory effect on OC formation and function in vitro. At concentrations of 6.25–25 *μ*M, there was no cytotoxic effect observed; osteoclastogenesis was significantly inhibited; and the expression of the OC-specific genes, including NFATC1, c-Fos, TRAP, CTR, CTSK, and V-ATPase d2, was suppressed. Further, OC differentiation was inhibited. Western blotting revealed that OST inhibited RANKL-induced P38/MAPK and NF-*κ*B signaling. The RANKL-induced signaling pathways, including the NF-*κ*B and p38 pathways, are generally critical for OC differentiation and survival [31]. There is accumulating evidence that the P38/MAPK pathway plays a key role in activating OC differentiation [32]. In our study, OST was inhibited by reducing the phosphorylation of P38 and P65, which contributed to the inhibition of RANKL-induced OC differentiation. Furthermore, NFATc1 is a master transcription factor that is closely regulated by NF-*κ*B activity [33]. Therefore, we demonstrated that OST decreased RANKL-induced P38/MAPK and NF-*κ*B activities, thereby inhibiting OC differentiation.

Increasing evidence indicates that ROS production underpins various pathological conditions, such as inflammation and osteoporosis [34, 35]. An increase in ROS production can accelerate bone resorption by OCs, accelerate OB bone apoptosis, and cause an imbalance in bone metabolism. ROS enhances the activation of the NF-*κ*B and MAPK pathways and induces the expression of inflammatory genes and OC-related genes [36]. This experiment demonstrated that OST could reduce cell damage caused by oxidative stress, which is one of the mechanisms of its anti-inflammatory effect, and indirectly showed the inhibitory effect of OST on OCs.

To ascertain the beneficial effects of OST in the treatment of these conditions, we used LPS, a potent stimulator, to induce inflammatory bone loss via the recruitment and activation of OC bone resorption [37].

Consistent with our in vitro findings, OST significantly reduced OCs in the bone tissue, indicating that OC recruitment and formation were inhibited. OST reduced the proportion of M1 macrophages and increased that of M2 macrophages at the site of inflammatory osteolysis. Despite the promising effects of OST on OC activity, there are some limitations to this study. Bone remodeling is maintained in vivo through OB formation and OC resorption. The effects of OST on OB and OC biology and function must be investigated to determine any deleterious effects on these cells. In addition, OST weakened the inflammatory effect of the M1 macrophages and promoted the anti-inflammatory effect to some extent in our study. Further studies are needed to determine whether it affects M2 polarization.

In summary, OST can exert anti-inflammatory and antiosteolytic effects in vitro and in vivo by regulating the ratio of macrophages to inflammation and is a potential drug for the treatment of inflammatory osteolysis.

## Figures and Tables

**Table 1:** Primers for the inflammatory genes and their sequences.

Gene	Forward	Reverse
GAPDH	ACCCAGAAGACTGTGGATGG	CACATTGGGGGTAGGAACAC
iNOS	ACTCAGCCAAGCCCTCACCTAC	TCCAATCTCTGCCTATCCGTCTCG
CCR7	TGTACGAGTCGGTGTGCTTC	GGTAGGTATCCGTCATGGTCTTG
TNF-*α*	GCCTCTTCTCATTCCTGCTTGTGG	GTGGTTTGTGAGTGTGAGGGTCTG
IL-6	CTTCTTGGGACTGATGCTGGTGAC	AGGTCTGTTGGGAGTGGTATCCTC
IL-10	GCTCTTACTGACTGGCATGAG	CGCAGCTCTAGGAGCATGTG
Arg-1	GGCAACCTGTGTCCTTTCTCCTG	GGTCTACGTCTCGCAAGCCAATG

**Table 2:** Genes and their sequences.

Gene	Forward	Reverse
GAPDH	ACCCAGAAGACTGTGGATGG	CACATTGGGGGTAGGAACAC
NFATc1	TGCTCCTCCTCCTGCTGCTC	GCAGAAGGTGGAGGTGCAC
CTR	TGCAGACAACTCTTGGTTGG	TCGGTTTCTTCTCCTCTGGA
CTSK	CTTCCAATACGTGCAGCAGA	TCTTCAGGGCTTTCTCGTTC
V-ATPase D2	AAGCCTTTGTTTGACGCTGT	TTCGATGCCTCTGTGAGATG
TRAP	CTTCCAATACGTGCAGCAGA	CCCCAGAGACATGATGAAGTCA
DC-STAMP	AAAACCCTTGGGCTGTTCTT	AATCATGGACGACTCCTTGG

## Data Availability

The datasets generated or analyzed during this study are available in the [Baidu Netdisk] repository, link: https://pan.baidu.com/s/1pNDEcZk1LKLOfi_PBoX8Ig?pwd=z5l0.
